# Chimeric antigen receptor T cell (CAR-T) immunotherapy for solid tumors: lessons learned and strategies for moving forward

**DOI:** 10.1186/s13045-018-0568-6

**Published:** 2018-02-13

**Authors:** Jian Li, Wenwen Li, Kejia Huang, Yang Zhang, Gary Kupfer, Qi Zhao

**Affiliations:** 1 School of Medicine, Chengdu University, Chengdu, 610106 China; 2 UCL Institute of Ophthalmology, 11-43 Bath Street, London, EC1V 9EL UK; 3 Sichuan Industrial Institute of Antibiotics, Chengdu University, Chengdu, 610052 China; 4 Section of Hematology-Oncology, Department of Pediatrics, Yale University School of Medicine, New Haven, CT 06520 USA; 5 College of Pharmacy and Biological Engineering, Chengdu University, Chengdu, 610106 China

**Keywords:** CAR-T, Solid tumor, Tumor microenvironment, Antigen recognition specificity, Safety control

## Abstract

Recently, the US Food and Drug Administration (FDA) approved the first chimeric antigen receptor T cell (CAR-T) therapy for the treatment CD19-positive B cell acute lymphoblastic leukemia. While CAR-T has achieved remarkable success in the treatment of hematopoietic malignancies, whether it can benefit solid tumor patients to the same extent is still uncertain. Even though hundreds of clinical trials are undergoing exploring a variety of tumor-associated antigens (TAA), no such antigen with comparable properties like CD19 has yet been identified regarding solid tumors CAR-T immunotherapy. Inefficient T cell trafficking, immunosuppressive tumor microenvironment, suboptimal antigen recognition specificity, and lack of safety control are currently considered as the main obstacles in solid tumor CAR-T therapy. Here, we reviewed the solid tumor CAR-T clinical trials, emphasizing the studies with published results. We further discussed the challenges that CAR-T is facing for solid tumor treatment and proposed potential strategies to improve the efficacy of CAR-T as promising immunotherapy.

## Background

**Fig. 1:**
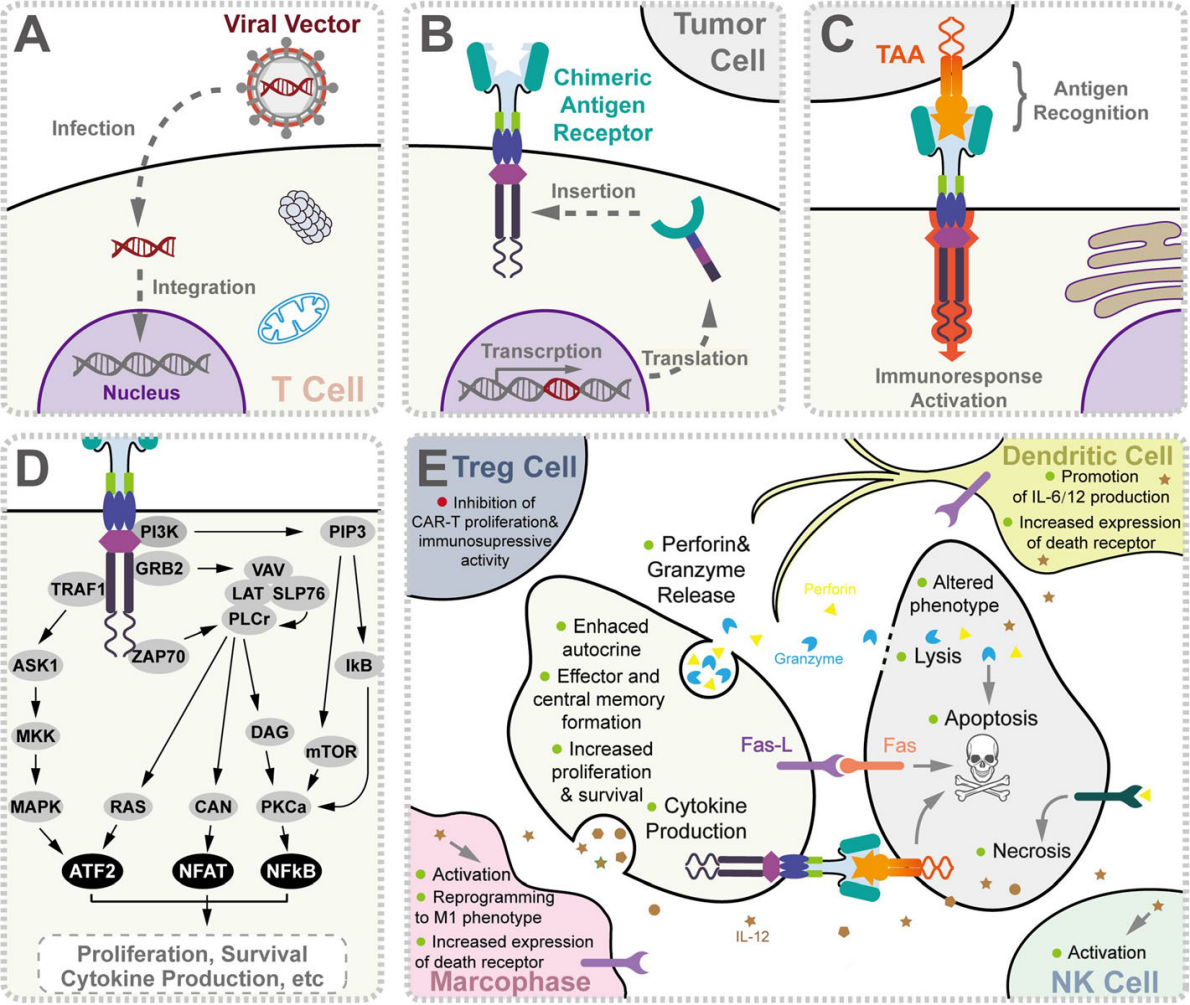
Molecular mechanism of chimeric antigen receptor T cell-mediated antitumor activity. **a** The chimeric T cell receptor coding sequence is delivered by viral vector. After entering into T cells (*beige*), virus was uncoated and transgene was preferably integrated at genome transcriptional start sites using specific vector designs, such as MLV retrovirus and piggyBac transposon. **b** CAR transgenes were endogenously transcript by host machinery, translated, and later inserted onto the T cell surface. **c** Association of CARs to TAA (*orange*) on tumor cell surface activates T cell for immunological response, for example, signaling network of CAR-T composed of CD8-CD28-CD137-CD3ζ domains was shown in (**d**). CAR-T-mediated immune response was reported to be amplified by ZAP70, TRAF1, PI3K, and GRB2 as well as other uncharacterized factors, giving rise to upregulation of signaling intermediates and subsequent pro-death gene transcriptions. **e** Upon CAR activation, T cells secreted cytokines (*brown*), perforins (*bright yellow*), and granzymes (*blue*) as well as activated death receptors, which triggered downstream targets. These subcellular events directly or subsequently contribute to specific death of tumor cells, including perforin and granzyme release, cytokine production, direct lysis, apoptosis, necrosis, reprogrammed phenotype, and immuno-memory formation in T cells, tumor cells (*gray*), macrophages (*pink*) (via IL-6, IL-10, IL-12, MCP-1, IP-10, TNF-α, MIP-1α, MIP-1β, IFN-γ), NK cells (*cyan*) (via IL-12, TNF-α, IFN-γ), Treg cells (*navy*) (via IL-2, IL-4, IL-7, IL-12, IL-15, IFN-β, IFN-γ, TSLP), and dendritic cells (*yellow*) (via IL-6, IL-10, IL-12, TNF-α, MIP-1α, MIP-1β, IFN-γ). Abbreviation: *NK cells* natural killer cells, *TAA* tumor-associated antigen, *Treg cells* regulatory T cells

Recently, the US Food and Drug Administration (FDA) approved the first chimeric antigen receptor T cell (CAR-T) therapy for the treatment of children and young adults with relapsed or refractory B cell acute lymphoblastic leukemia (ALL) positive for CD19 antigen [1, 2]. Chimeric antigen receptors (CARs) are chimeric immunoglobulin T cell receptor (TCR) molecules derived from transgenes encoding for single-chain variable fragments (scFv), which originate from antibodies capable of recognizing tumor-associated antigens (TAA) [3, 4]. Mechanistically, the CAR-T cell recognizes and binds to TAA, inducing a conformational change that transmits the binding signal into the CAR-T cell. Activation signal through the CD3ζ domain and costimulatory domains activate CAR-T cell, leading to cytokine release and transcription factor expression, which promote T cell survival and function and eventually induce cytotoxic activities against tumor cells [5, 6]. The molecular mechanisms of CAR-T immunotherapy were summarized in Fig. 1.

The designs of CARs are grouped schematically into three generations with increasing costimulatory activity [7]. The first generation CARs are conjugated with TCR-CD3ζ chain alone, which is capable of providing a comparable stimulatory signal to that of the entire CD3 complex [8, 9]. However, this CAR configuration is insufficient to prime resting T cells for proliferation and cytokine production, affecting sustained antitumor responses in vivo [10]. With the aim to enhance the stimulation effect, the second-generation CARs include a costimulatory module on the basis of the first generation, which was initially designed in the 1990s [8, 9, 11, 12]. CD28 is one of the most commonly utilized molecules for this purpose to promote interleukin-2 (IL-2) secretion and improve T cell activity [13–16]. On top of CD3ζ and co-stimulators like CD28, additional costimulatory domains, such as OX40 or 4-1BB, were added to the third generation CARs to further enhance the signaling capacity [17, 18]. The fourth-generation CARs added IL-12 to the base of the second-generation constructs, which are known as T cell redirected for universal cytokine-mediated killing (TRUCKs). TRUCKs augment T cell activation and activate and attract innate immune cells to eliminate antigen-negative cancer cells in the targeted lesion. Such TRUCK T cells can also treat viral infections, metabolic disorders, and auto-immune diseases [19–21].

Whereas ongoing CAR-T clinical trials for the treatment of leukemia and lymphoma have demonstrated durable remission of the disease or even cure, CAR-T therapy targeting solid tumor is still in an infant stage. One of the most frequently asked questions is whether CAR-T can benefit solid tumor patients to the same extent as it does for blood malignancies. Here, we reviewed the published results of clinical studies for solid tumor CAR-T treatment. We further discussed the challenges that CAR-T is facing for solid tumor treatment and proposed potential strategies to improve the efficacy of CAR-T as promising immunotherapy.

## Clinical trials using engineered CAR-T cells to treat solid tumor

Because of the success achieved in CAR-T therapy targeting B cell malignancies and the advancements in CAR-T preclinical studies for solid tumors, more than 100 CAR-T clinical trials targeting solid tumors have been initiated at medical centers all over the world (Table 1).

**Table 1:** Selected CAR-T clinical trials targeting solid tumor-associated antigens

Antigen	CAR design	Identifier	Disease condition	Phase	Status	Sponsor	Notes	Ref
FR-α	1st (FcRγ)	NCT00019136	Ovarian cancer	I	Completed	National Cancer Institute		[35]
	4th	NCT03185468	Bladder cancer, urothelial carcinoma bladder	I/II	Recruiting	Shenzhen Geno-immune Medical Institute		
CAIX	1st	DDHK97-29/P00.0040C	Renal cell carcinoma	I/II	Completed	Erasmus University Medical Center	First clinical study of its nature in Europe	[36, 115–117]
L1-CAM	2nd and 3	NCT02311621	Neuroblastoma	I	Recruiting	Seattle Children’s Hospital		
	1st	NCT00006480	Neuroblastoma	I	Completed	Fred Hutchinson Cancer Center		[38]
MSLN	NA	NCT01583686	Cervical, pancreatic, ovarian, and lung cancer, mesothelioma	I/II	Recruiting	National Cancer Institute		[118]
	2nd	NCT01355965	Malignant pleural mesothelioma	I	Completed	University of Pennsylvania	Intravenous (IV) administration of RNA mesothelin redirected autologous T cell	[39, 40]
	2nd	NCT02159716	Pancreatic, ovarian cancer, malignant epithelial pleural mesothelioma	I	Completed	University of Pennsylvania		
	2nd	NCT01897415	Metastatic pancreatic ductal adenocarcinoma	I	Completed	University of Pennsylvania	Intravenous (IV) administration of RNA mesothelin redirected autologous T cell	[41]
	2nd	NCT02465983	Pancreatic cancer	I	Completed	University of Pennsylvania		
	2nd	NCT02414269	Mesothelioma, lung and breast cancer	I	Recruiting	MSKCC	iCasp9M28z T cell	
	2nd	NCT02706782	Pancreatic cancer	I	Recruiting	Shanghai GeneChem Co., Ltd.	Vascular intervention-mediated CAR-T infusion	
	NA	NCT03030001	Solid tumors	I/II	Recruiting	Ningbo Cancer Hospital	PD-1 antibody expressing mesothelin-specific CAR-T cells	
	NA	NCT03182803	Advanced solid tumors	I/II	Recruiting	Shanghai Cell Therapy Research Institute	CTLA-4/PD-1 antibodies expressing mesoCAR-T	
HER2	NA	NCT00924287	HER2-positive sarcoma	I/II	Terminated	National Cancer Institute		[21, 119]
	2nd	NCT00902044	HER2-positive sarcoma	I/II	Recruiting	Baylor College of Medicine		[28]
	NA	NCT01935843	HER-2 positive solid tumors	I/II	Recruiting	Chinese PLA General Hospital		[42]
	2nd	NCT02547961	Breast cancer	I/II	Completed	Fuda Cancer Hospital		
	2nd	NCT02442297	Glioblastoma	I	Recruiting	Baylor College of Medicine		
	2nd	NCT01109095	Glioblastoma multiforme	I	Ongoing	Baylor College of Medicine	Genetically modified HER.CAR CMV-specific CTLs	[43]
	2nd	NCT00889954	HER2 positive malignancies	I	Ongoing	Baylor College of Medicine	TGFbeta dominant-negative receptor (DNR) expressing EBV specific lymphocytes	
EGFR	2nd	NCT01869166	Advanced EGFR-positive solid tumors	I/II	Recruiting	Chinese PLA General Hospital		[44]
	NA	NCT03182816	Advanced solid tumor	I/II	Recruiting	Shanghai Cell Therapy Research Institute	Anti-CTLA-4/PD-1 expressing EGFR-CAR-T	
	NA	NCT02873390	Advanced solid tumor		Recruiting	Ningbo Cancer Hospital	PD-1 antibody expressing CAR-T cells	
	NA	NCT02862028	Advanced solid tumor	I/II	Recruiting	Shanghai International Medical Center	PD-1 antibody expressing CAR-T cells	
EGFRvIII	3rd	NCT01454596	Glioma, glioblastoma, brain cancer	I/II	Recruiting	National Cancer Institute		[120]
	NA	NCT02209376	EGFRvIII positive glioma	I	Ongoing	University of Pennsylvania		
		NCT03170141	Glioblastoma multiforme	I/II	Recruiting	Shenzhen Geno-immune Medical Institute	4SCAR-IgT cells producing PD1 and PD-L1 antibodies	
IL13Rα2	1st	NCT00730613	Glioblastoma	I	Completed	City of Hope Medical Center	CAR expresses the Hy/TK selection/suicide fusion protein	[30]
	2nd	NCT02208362	Glioblastoma	I	Recruiting	City of Hope Medical Center	Hinge-optimized	
	1st	NCT01082926	Glioblastoma	I	Completed	City of Hope Medical Center	T cells modified to express HyTK and to be resistant to glucocorticoids, in combination with interleukin-2	
CEA	2nd	NCT01373047	Liver metastases	I	Completed	Roger Williams MC		[31, 121]
	2nd	NCT02416466	Liver metastases	I	Ongoing	Roger Williams MC		[122]
	2nd	NCT02349724	Lung, colorectal, gastric, breast, and pancreatic cancer	I	Recruiting	Southwest Hospital		[123]
	2nd	NCT02862704	Liver metastases	I/II	Recruiting	Xijing Hospital	MG7 specific CAR-T, which is a glycosylated protein of CEA	
	NA	NCT02850536	Liver metastases	I	Recruiting	Roger Williams MC	delivered via the hepatic artery using the Surefire Infusion System (SIS)	
	NA	NCT00004178	Solid tumor	I	Completed	Roger Williams MC		
	1st	NCT01212887	Adult solid tumor,	I	Terminated	Cancer Research UK	MFE23 scFv-expressing autologous anti-CEA MFEz T lymphocytes	
GD2	1st	NCT00085930	Neuroblastoma	I	Ongoing	Baylor College of Medicine	14g2a.zeta chimeric receptor-transduced autologous EBV specific cytotoxic T lymphocytes (EBV-CTL) w/o lymphodepletion	[46, 124]
	3rd	NCT01822652	Neuroblastoma	I	Ongoing	Baylor College of Medicine	iC9-GD2-CD28-OX40 (iC9-GD2) T cells	[125]
	3rd	NCT01953900	Sarcomas	I	Ongoing	Baylor College of Medicine		[126]
	3rd	NCT02439788	Neuroblastoma	I	Withdrawn	Baylor College of Medicine	3rd generation GD2 specific CAR and inducible caspase 9 safety switch transduced autologous natural killer T cells	
	NA	NCT01460901	Neuroblastoma	I	Completed	Children’s Mercy Hospital, Kansas		[125]
	3rd	NCT02107963	Osteosarcoma, neuroblastoma, Melanoma	I	Completed	National Cancer Institute		[127]
	4th	NCT02765243	Neuroblastoma, effects of immunotherapy	II	Recruiting	Zhujiang Hospital		
	4th	NCT02992210	Solid tumor	I/II	Recruiting	Shenzhen Geno-immune Medical Institute	4th generation CAR (4SCAR) fused with an inducible apoptotic caspase 9 domain	
MUC1	NA	NCT02617134	Glioma, colorectal and gastric carcinoma	I/II	Recruiting	PersonGen BioTherapeutics		
	3rd	NCT02587689	NSCLC, hepatocellular, pancreatic, and breast carcinoma	I/II	Recruiting	PersonGen BioTherapeutics		[29]
	NA	NCT03179007	Advanced solid tumor		Recruiting	Ningbo Cancer Hospital	Anti-CTLA-4/PD-1 expressing MUC1-CAR-T	
PSMA	2nd	NCT01140373	Prostate cancer	I	Ongoing	MSKCC		
	NA	NCT01929239	Prostate cancer	I/II	Suspended (funding)	Roger Williams MC		[128]
	1st	BB-1ND12084	Prostate cancer	I		Roger Williams MC		[48]
	4th	NCT03185468	Bladder cancer, urothelial carcinoma bladder	I/II	Recruiting	Shenzhen Geno-immune Medical Institute		
PSCA	NA	NCT03198052	Lung cancer	I	Recruiting	Guangzhou Medical University		
	NA	NCT02744287	Pancreatic cancer	I	Recruiting	Bellicum Pharmaceuticals		
FAP	2nd	NCT01722149	Malignant pleural mesothelioma	I	Recruiting	University of Zurich		[32]
CD133	1st, 2nd	NCT02541370	CD133-positive malignancies	I	Recruiting	Chinese PLA General Hospital		[45]
cMet	NA	NCT01837602	Breast	I	Ongoing	University of Pennsylvania	cMet RNA CAR-T cells	[47]
	2nd	NCT03060356	Malignant melanoma, breast cancer	I	Recruiting	University of Pennsylvania	cMet RNA CAR-T cells	
EphA2	NA	NCT02575261	Glioma	I/II	Completed	Fuda Cancer Hospital		
GPC3	2nd	NCT02715362	Hepatic carcinoma	I/II	Recruiting	Shanghai GeneChem Co., Ltd.	Administration by transcatheter arterial infusion (TAI) method	
		NCT02723942	Hepatocellular carcinoma	I/II	Completed	Fuda Cancer Hospital, Guangzhou		
		NCT02876978	Lung squamous cell carcinoma	I	Recruiting	Carsgen Therapeutics, Ltd.		
		NCT03130712	Hepatocellular carcinoma	I/II	Recruiting	Shanghai GeneChem Co., Ltd.		
	3rd	NCT03198546	Hepatocellular carcinoma	I	Recruiting	Guangzhou Medical University		
VEGFR-II	NA	NCT01218867	Melanoma, renal cancer	I/II	Completed	National Cancer Institute		
ROR1	NA	NCT02706392	Breast, lung carcinoma	I	Recruiting	Fred Hutchinson Cancer Center		[129]
EpCAM	NA	NCT02729493	Liver neoplasms		Recruiting	Sinobioway Cell Therapy Co., Ltd.		
	NA	NCT02725125	Stomach neoplasms		Recruiting	Sinobioway Cell Therapy Co., Ltd.		
	2nd	NCT02915445	Malignant neoplasm of nasopharynx, breast Cancer	I	Recruiting	Sichuan University		
	2nd	NCT03013712	Solid tumors	I/II	Recruiting	First Affiliated Hospital of Chengdu Medical College		
MUC16ecto	2nd	NCT02498912	Solid tumors	I	Recruiting	MSKCC	T cells genetically engineered to secrete IL-12	[130]

Representative clinical trials using CAR-T to treat solid tumors are grouped based on the tumor-associated antigens they are targeting. Trial-related information were mainly collected from published results and *ClinicalTrials.gov*

Abbreviations: *CAIX* carboxyanhydrase-IX, *CEA* carcinoembryonic antigen, *cMET* hepatocyte growth factor receptor, *EGFR* epidermal growth factor receptor, *EpCAM* epithelial cell adhesion molecule, *EphA2* EPH receptor A2, *FAP* fibroblast activation protein α, *GD2* disialoganglioside, *GPC3* glypican-3, *HER2* human epidermal growth factor receptor-2, *L1-CAM* L1 cell adhesion molecule, *MSLN* mesothelin, *MUC1* mucin, *NA* not applicable, *PSMA* prostate-specific membrane antigen, *ROR1* receptor tyrosine kinase-like orphan receptor 1, *VEGFR* vascular endothelial growth factor receptor

### Tumor-associated antigens and CAR design

**Fig. 2:**
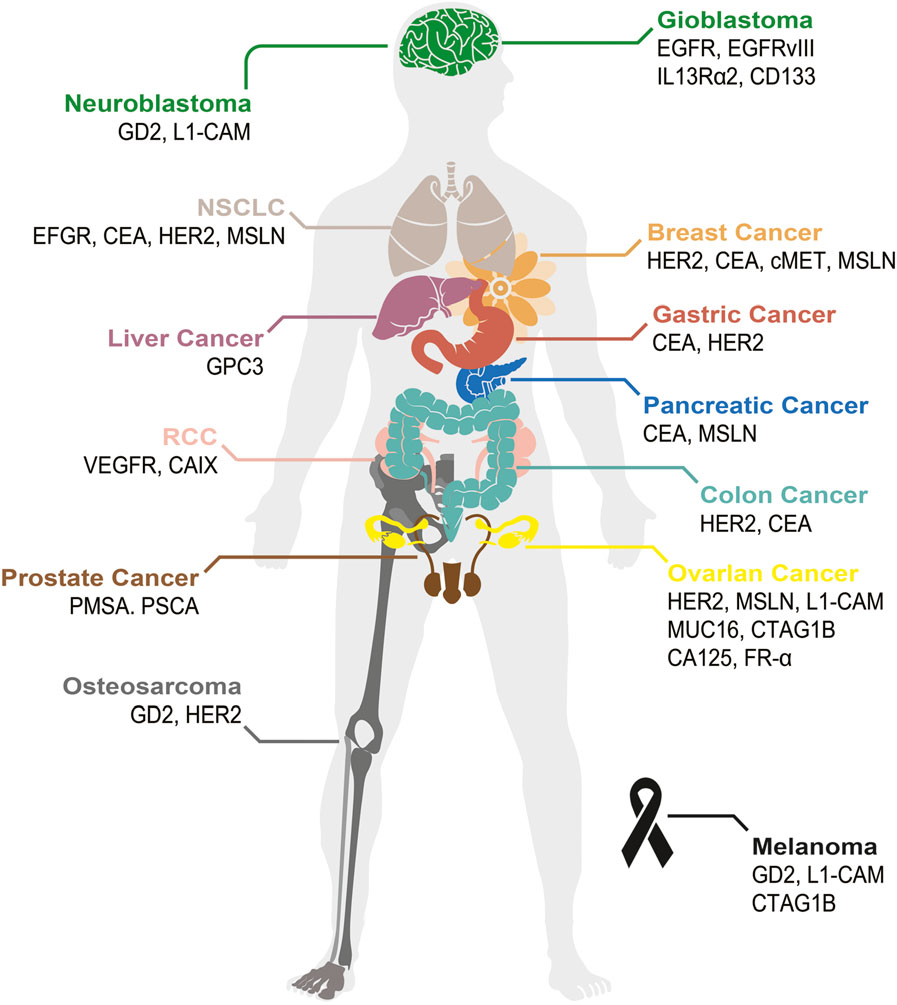
Tumor-associated antigens targeted in CAR-T therapy. Schematic illustration of a human body whose tissues or organs have been investigated in preclinical and clinical studies for solid tumor immunotherapy using CAR-T, including brain (*green*), lungs (*beige*), mammary gland (*orange*), liver (*purple*), stomach (*red*), pancreas (*blue*), kidneys (*pink*), colon (*cyan*), male reproductive system (*brown*), ovary (*yellow*), and bones (*gray*) as well as skin (*black*). Abbreviation: *CCA* cholangiocarcinoma, *MPM* malignant pleural mesothelioma, *NSCLC* non-small-cell lung carcinoma, *RCC* renal cell carcinoma

So far, no such cell surface antigen with comparable properties as CD19 has yet been identified regarding solid tumors. An ideal molecule for CAR targeting should be overexpressed on cancer cell surface of many patients, with zero or very low expression in normal tissues. Currently, TAAs, including mesothelin (MSLN), HER2, EGFR/EGFRvIII, GD2, CEA, IL13Rα2, MUC1, FAP, PSMA, and PSCA, are extensively investigated in CAR-T therapy for solid tumors [22, 23]. TAAs currently being exploited for CAR-T therapy in solid tumors are summarized (Fig. 2). Yu and colleagues comprehensively discussed these antigens regarding their biological functions and antitumor activities [22]. As shown in Table 1, most of the solid tumor CAR-T clinical trials utilize the second or third generation of CARs, which contain either CD28 alone or CD28-4-BB1/OX40 as the costimulatory signal. Notably, a few of these studies, e.g., the trials targeting GD2 (NCT02765243, NCT02992210), PMSA (NCT03185468), FR-α (NCT03185468), investigated the efficacy of the fourth-generation CARs, i.e., TRUCK, which includes a transgenic cytokine expression cassette in the CAR constructs [24]. Because of the tremendous phenotypic diversity in solid tumor lesions, a reasonable number of cancer cells are not recognized by a given CAR. The introduction of a transgenic cytokine such as interleukin-12 (IL-12) initiates universal cytokine-mediated killing towards those cancer cells that are invisible to CAR-T cells [19].

CAR-T cell therapy must carefully balance efficient T cell activation, to ensure antitumor activity, with the potential for uncontrolled cytotoxicity. Some recent clinical trials included an inducible caspase 9 (iCasp9) “safety switch” in their CAR construct, which allows for the removal of inappropriately activated CAR-T cells [25, 26]. The administration of the small molecule drug AP1903 causes the dimerization and activation of iCasp9, resulting in rapid induction of apoptosis in transduced cells. Two CAR-T clinical trials targeting GD2 performed at Baylor College of Medicine incorporated such iCasp9 switch into their third generation CAR constructs (NCT01822652, NCT02439788). In another recently initiated trial led by Chang, a fourth-generation CAR was fused with the iCasp9 domain (NCT02992210).

PD-L1 expression in solid tumors counteracts the efficacy of CAR-T cell [27]. To overcome the inhibitory effect of PD-LI expression on CAR-T therapy, some clinical trials (e.g., NCT03030001, NCT03182803-MSLN, NCT03182816, NCT02873390, NCT02862028-EGFR, NCT03170141-EGFRvIII, and NCT03179007-MUC1) add PD-1 dominant-negative receptor expressing gene to CAR-T cells, providing cell-intrinsic checkpoint blockade and increase antitumor efficacy. Based on the currently available data, anti-PD-1 combination therapy approach may be useful to augment CAR-T cell efficacy and persistence in patients [27].

### T cell dosage, administration, and persistence

According to the published results and clinical trial description available on the website, the majority of the CAR-T clinical trials use a dose escalation regime, which usually covers two log steps with a starting point ranged from around 1 × 10^6^ and 1 × 10^9^ CAR-T cells [24]. It is important to keep in mind that the percentage of CAR-positive T cells varies significantly not only among different trials but also among different batches within single trial [24]. One of the major sources for such variation is the different production procedure of CAR-T cells adopted by different trials, emphasizing the importance of standardizing the CAR-T cells production protocol. Notably, to increase the tolerability of the treatment and to lower the risk of side effects, the given CAR-T cell dose is often split over multiple injections. For example, Ahmed and colleagues reported administration of 1 × 10^4^–1 × 10^8^/m2 HER2 specific CAR-T cells in up to 9 infusions (NCT00902044) [28]. Typically, CAR-T cells are infused intravenously. However, intratumoral (NCT02587689) [29], intracranial (NCT00730613) [30], hepatic artery (NCT01373047) [31], and pleural [32] administration are being investigated as well. The CAR-T cell administration method could have a dramatic impact on the efficacy of the treatment. Brown et al. compared the effectiveness of two intracranial CAR-T cell delivery route-infusion into the resected tumor cavity and infusion into the ventricular system [33]. While intracavitary therapy appeared to control local tumor recurrence, progression of glioblastoma at distant sites was observed. By contrast, intraventricular administration of CAR-T cells leads to the regression of all central nervous system tumors, including spinal tumors. After the administration, CAR-T cell persistence is a key factor that determines the efficacy of the therapy. One major problem of current CAR-T cell immunotherapy is that T lymphocytes have limited replicative lifespans [34], which potentially influences the long-term antitumor effect of CAR-T cell immunotherapy. The reported T cell persistence in published clinical trials ranged from up to 4 weeks (DDHK97-29/P00.0040C, BB-IND 12084) to up to 192 weeks (NCT00085930).

### Clinical outcomes and toxicities

Some early attempts using CAR-T to treat solid tumors were not successful. In 2006, Kershaw and colleagues tested the efficacy of CAR-T cells targeting ovarian cancer cells expressing alpha-folate receptor (FR-α) (NCT00019136) [35]. Even though the patients can tolerate the administration of FR-α specific CAR-T cells, no clinical response was observed in all the patients (14 out of 14), likely because of the inefficient localization and short-term persistence of the CAR-T cells [35]. Lamers and colleagues treated 12 patients with CAIX-expressing metastatic renal cell carcinoma with CAIX specific CAR-T cells [36, 37]. Unfortunately, no clinical responses were observed, and some patients developed anti-CAR-T cell antibodies and severe live enzyme disturbance.

Park et al. reported treating metastatic neuroblastoma with L1-CAM specific CAR-T cells at a dosage of 1 × 10^8^ cells/m^2^ to 1 × 10^9^ cells/m^2^ (NCT00006480) [38]. The persistence of cells was measured to be 1–7 days for patients with heavy disease burden and 42 days for a patient with light disease burden. One of the six enrolled patients achieved stable disease after treatment [38]. A series of CAR-T clinical trials targeting MSLN have been performed in many different types of solid tumors, such as mesothelioma (NCT01355965), pancreatic cancer (NCT01583686, NCT02465983, NCT02706782), and breast cancer (NCT02792114). In one of these trails, advanced mesothelioma patients were administered autologous T cells electroporated with the mRNA encoding for MSLN CAR (NCT01355965) [39, 40]. Moderate clinical responses were observed as supported by the detection of MSLN specific CAR-T cells in the tumor site and the transient elevation of inflammatory cytokines [39, 40]. MSLN CAR T cell infusions were well tolerated at the dosage tested without severe toxicities [39, 40]. Unfortunately, one of the study subjects was reported to develop severe anaphylaxis and cardiac arrest after the third infusion of MSLN CAR-T cells [40], which is likely to result from the inclusion of the murine SS1 scFv in the CAR-T design. In another trial for pancreatic ductal adenocarcinoma (PDAC) (NCT01897415), Beatty and colleagues utilized mRNA-encoded mesothelin-specific CAR to treat six patients, in which the infusion were well tolerated and preliminary evidence of antitumor efficacy was observed, supported by stable disease seen in two treated patients [41].

Clinically, the safety and efficacy of HER2-specific CAR-T cells in patients with relapsed/refractory HER2-positive sarcoma has been evaluated in a phase I/II clinical study (NCT00902044, NCT01109095, and NCT00924287). In a dose escalating trial (NCT00902044, 1 × 10^4^/m^2^ to 1 × 10^8^/m^2^), 4 out of 19 subjects acquired stable disease [28]. Feng et al. performed a phase I clinical trial investigating HER2-specific CAR-T cells in patients with advanced biliary tract cancers (BTCs) and pancreatic cancers (PCs) (NCT01935843). Among the 6 patients received HER2-specific CAR-T infusion, one patient obtained a 4.5-month partial response and the other 5 patients achieved stable disease with mild to moderate adverse events [42]. In another trial (NCT01109095), 17 CMV-seropositive patients with radiologically progressive HER2-positive glioblastoma were infused with HER2 CMV bispecific CAR-T cells at the dose of 1 × 10^6^/m^2^–1 × 10^8^/m^2^ [43]. Such treatments were well tolerated without severe adverse events or cytokine release syndrome, and 7 out the 17 treated patients achieved stable disease [43].

Feng and colleagues reported the clinical trials of EGFR specific CAR-T treating non-small cell lung cancer (NCT01869166) [44]. Five out of the 11 treated patients achieved stable disease and 2 achieved partial response. The CAR-T infusion-related adverse events were mild and manageable. Recently, Feng et al. reported treating a patient diagnosed as advanced unresectable/metastatic cholangiocarcinoma (CCA) with CAR-T cocktail immunotherapy, which was composed of successive infusions of CAR-T cells targeting epidermal growth factor receptor (EGFR) and CD133 (NCT01869166, NCT02541370) [45]. The patient achieved an 8.5-month partial response (PR) from the CART-EGFR therapy and a 4.5-month-lasting PR from the CART133 treatment. However, a series of adverse events were also observed in the course of treatment, including deteriorative grade 3 systemic subcutaneous hemorrhages and congestive rashes together with serum cytokine release.

In 2015, Brown et al. reported that three patients with recurrent glioblastoma were treated with CAR-T cells targeting IL13Rα2 (NCT00730613) [30]. Patients received up to 12 local infusions at a maximum dose of 1 × 10^8^ CAR-T cells. Evidence for transient antitumor activity was observed in two of the patients with manageable temporary central nerve system inflammation. The same group conducted another clinical trial (NCT02208362), in which tumor regression and increased production of cytokines and immune cells were observed [33]. Encouragingly, the clinical response continued for 7.5 months after the initiation of CAR-T cell therapy, indicating a relatively long persistence of CAR-T cells.

Katz and colleagues conducted a phase I trial to test CAR-T in patients with CEA-positive liver metastases (NCT01373047). Among the six patients who completed the protocol, one patient remained alive with stable disease at 23 months following CAR-T treatment and five patients died of progressive disease [31]. Biopsies demonstrated an increase in liver metastases necrosis or fibrosis in four out of six patients. No patient suffered a grade 3 or 4 adverse event related to the CAR-T treatment. Louis and colleagues evaluated the efficacy of GD2-specific CAR-T in 19 patients either with remission (8 patients) or progressive neuroblastoma (11 patients) (NCT00085930) [46]. Three of 11 patients with active disease achieved complete remission and up to 192 weeks of CAR-T cell persistence was observed in the trial [46].

Clinical trials investigating the efficacy of CAR-T cells targeting MUC1 (NCT02587689) [29], cMet (NCT01837602, NCT03060356) [47], PSMA (BB-IND 12084) [48], VEGFR-2 (NCT01218867) have been reported. Relevant information and results of CAR-T clinical trials were summarized in Table 2.

**Table 2:** CAR-T clinical trials for solid tumors with published results

Antigen	Identifier	Patients *n* Age (media*n*; range)	Dosage	Persistence	Outcome *n* (median; range) in month	Adverse events	Ref
FR-a	NCT00019136	14 (33–60)	0.3–5 × 10^10^ T cells	Up to > 1 year	NE: 14	Grade 3/4 toxicities including hypotension and dyspnea as well as less frequently fatigue, leukopenia, rigors, sinus tachycardia, and diarrhea, in some patients receiving IL-2	[35]
CAIX	DDHK9729/P00.0040C	12 62,5 (46–74)	0.2–2.1 × 10^9^ (split dose over 2 × 5 days)	Up to 4 weeks	NE: 12	Transient liver enzyme disturbances (grade 3/4) observed in 4 patients, caused by attack of CAR-T cells to CAIX-expressing bile duct epithelial cells	[36, 115–117]
L1-CAM	NCT00006480	6 9,5 (7–16)	1 × 10^8^–1.1 × 10^9^/m^2^ (split dose; 2–3 times of infusions 14 days apart)	Up to 42 days	CR: 1 (1,5) SD: 1 (1) PD: 4	Grade 3 lymphopenia, neutropenia, low hemoglobin and bacteremia caused by 10^8^/m^2^ CAR-T cells infusion grade 3 pneumonitis for one patient, which was associated with 10^9^/m^2^ CAR-T cells infusion	[38]
MSLN	NCT01355965	2 78 (75–81)	3 infusions with 0.1–1 × 10^9^ cells or 8 infusions with 3 × 10^8^ cells and 2 infusions with 2 × 10^8^ cells	Transient (mRNA CAR)	PR: 1 (6) SD: 1	Anaphylactic reaction in one patient, leading to grade 4 cardiac arrest, respiratory failure, disseminated intravenous coagulation, and CRS grade 4 jejunal obstruction, grade 3 abdominal pain, and grade 2 lymphcytosis for another patient	[39, 40]
	NCT01897415	NA	3 times per week for 3 weeks	Transient (mRNA CAR)	SD: 2	Grade > 3 toxicities included abdominal pain (1) and back pain (1)	[41]
HER2	NCT00924287	1 39	1 × 10^10^ total	NA	Death: 1	Respiratory distress and dramatic pulmonary infiltrate on chest X-ray were observed soon after CAR-T cell administration. Severe hypotension, bradycardia, gastrointestinal bleeding, resulting in a cardiac arrest. The patient died 5 days after the CAR-T infusion.	[119]
	NCT01109095	17 49 (11–79)	1 × 10^6^-1 × 10^8^/m^2^	Up to 12 weeks	SD: 7 PD: 8 PR: 1	NA	[43]
	NCT00902044	19 16 (7–29)	1 × 10^4^-1 × 10^8^/m^2^ (up to 9 infusions)	Up to 18 month	SD: 4 (0,5; 0,5–14) PD: 13 NE: 2	Fever observed in 1 patient	[28]
	NCT01935843	11 61 (50–75)	2.1 × 10^6^/kg (range 1.4–3.8 × 10^6^/kg), 1 to 2 cycles	Up to 80 days	PR: 1 (4.5) SD: 5 (4.8; 1.5–8.3)	Mild-to-moderate fatigue, nausea, vomiting, myalgia, arthralgia, and lymphopenia, Except one grade 3 acute febrile syndrome and one abnormal elevation of transaminase	[42]
EGFR	NCT01869166	11 58 (40–66)	4 × 10^5^–2.54 × 10^7^/kg	Up to 37 weeks	PR: 2 (3; 2–3,5) SD: 5 (5,5; 2–8+) PD: 4	Mild skin toxicity, nausea, vomiting, dyspnea, and hypotension; one patient suffered from a transient grade 3/4 increase in serum lipase	[44]
EGFR and CD133	NCT01869166 NCT02541370	1 52	EGFR: 2.2 × 10^6^/kg first cycle, 2.1 × 10^6^/kg second cycle, CD133: 1.22 × 106/kg	NA	EGDR: PR (8.5) CD133: PR (2.5)	EGFR: mild chills, fever, fatigue, vomiting and muscle soreness, and a 9-day duration of delayed lower fever CD133: an intermittent upper abdominal dull pain, chills, fever, and rapidly deteriorative grade 3 systemic subcutaneous hemorrhages and congestive rashes together with serum cytokine release	[45]
IL-13Ra2	NCT00730613	3 57 (36–57)	1.6 × 10^8^ for first cycle + 3 × 10^8^ for second to fourth cycle (split dose in each cycle; 3 infusions 2 days apart)	Up to 14 weeks	PR: 2 (12; 10–14) PD: 1	Grade 3 headache in two patients receiving 10^8^ CAR-T cells infusion grade 3 neurologic adverse events observed in 1 patient	[30]
	NCT02208362	1 50	Intracavitary infusions (cycles 1 through 6) and intraventricular infusions (cycles 7 through 16), maximum dose: 10 × 10^6^ cells	Detectable at 149 days after enrollment	CR:7.5	No CAR-T cell infusion related toxic effects of grade 3 or higher were observed	[33]
CEA	NCT01373047	7 53,5 (51–66)	1 × 10^8^–1 × 10^10^ cells (single hepatic artery infusion)	NA	SD: 1 (23) PD: 5 NE: 1	Grade 3 fever and tachycardia (1 patient), associated to high-dose IL-2 administration. grade 1/2 transient elevations of alkaline phosphatase, total bilirubin and aspartate aminotransferase levels observed in all patients	[31]
GD2	NCT00085930	19 7 (3–29)	1.2 × 10^7^-1 × 10^8^/m^2^ (single infusion)	Up to 192 weeks for ATC and 96 weeks for CTLs	CR: 11 PR: 1 SD: 1 PD: 6	Mild to moderate local pain at the site of tumor necrosis in two patients	[46, 124]
MUC1	NCT02587689	1	5 × 10^5^ cells per lesion	NA	PR: 1	Mild headache, muscle pain, nasal congestion, and abdominal bloating discomfort, and a transient CRS was experienced	[29]
PSMA	BB-IND 12084	5 61 (51–75)	1 × 10^9^–1 × 10^10^ cells	Up to 4 week	PR: 3 NE: 2	Grade 3/4 hematologic toxicities including neutropenia, neutropenic fever, and thrombocytopenia in all the patients; anemia, hypocalcemia, hypophosphatemia, and appendicitis in one patient. Grade 1/2 skin rash, fatigue, intermittent low-grade fevers, and muscle pain in some patients	[48]
VEGFR-II	NCT01218867	23	1 × 10^6^–3 × 10^10^ cells	NA	PR: 1 SD: 1 PD: 21	Grade 3/4 toxicity include nausea, vomiting, hypoxia, and elevated levels of aspartate transaminase, alanine transaminase, and bilirubin	Results available at www.ClinicalTrials.gov

The patient characteristics, CAR-T treatment dosage, CAR-T cell persistence, clinical outcomes, and adverse events of CAR-T clinical trials with published results are listed

Abbreviations: *NE* no response, *CR* clinical response, *PR* partial response, *SD* stable disease, *PD* progress disease, *NA* not available

As learned from the results of these clinical trials, CAR-T cells face a unique set of challenges in the case of solid tumors. Some of the issues appear to be the absence of unique tumor-associated antigens, the inefficient homing of T cells to tumor sites, and the limited persistence of CAR-T cells. Moreover, the immunosuppressive microenvironment within the tumor tends to inhibit CAR-T cell function strongly. While a seemingly complicated, fulfilling all of above requirements can be accomplished efficiently through both intrinsic and/or extrinsic modifications of CAR-T cells.

## Overcoming challenges with smarter CAR designs

**Fig. 3:**
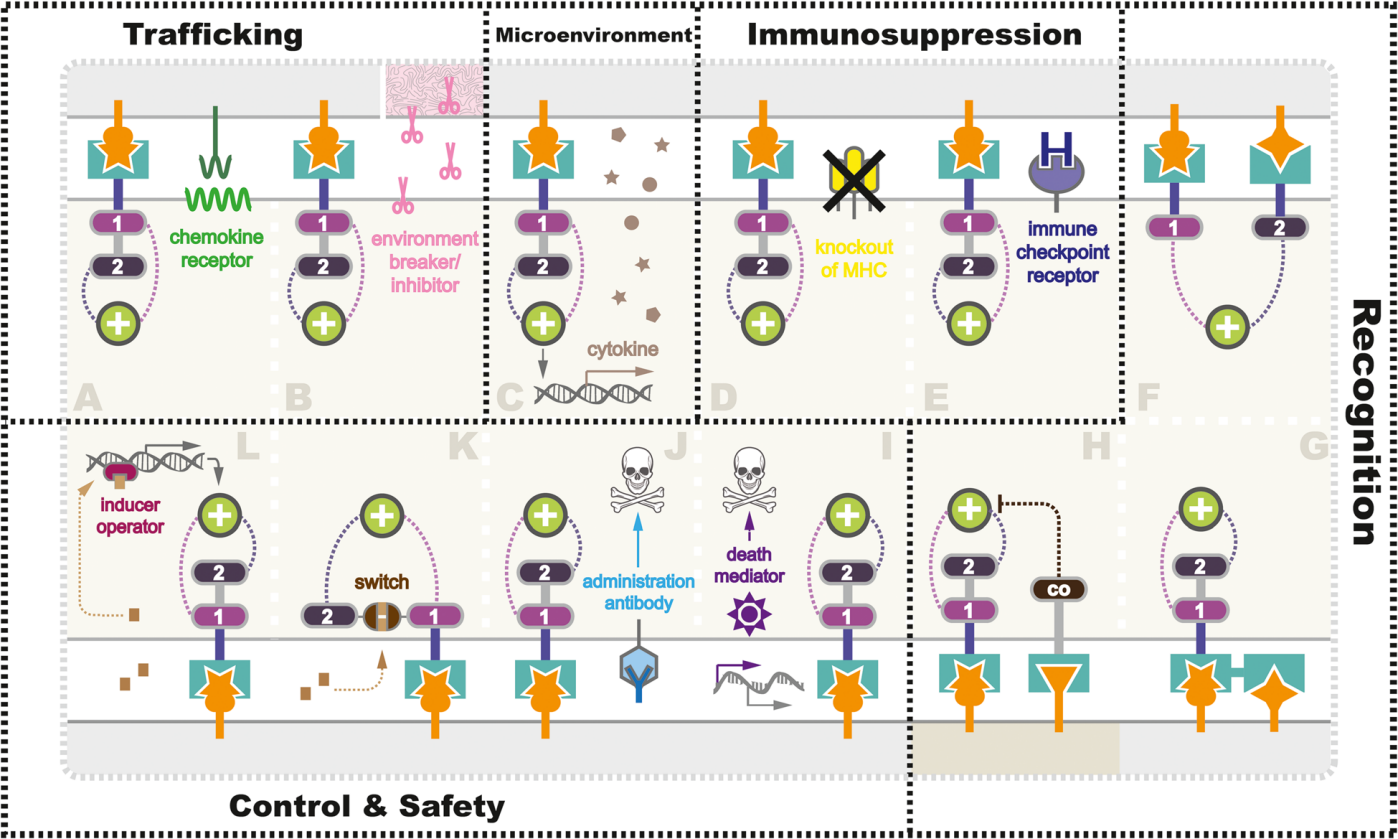
Strategies being exploited to overcome challenges in CAR-T therapy in solid tumor. Various strategies are currently being tested in preclinical and clinical studies to overcome the challenges facing CAR-T therapy for solid tumor (*gray*), including **a**, **b** enhancing T cell (*beige*) trafficking, **c** reforming tumor microenvironment (*pink represented physiological barriers*), **d**, **e** anti-immunosuppression, elevating antigen recognition towards tumor (**f**, **g**), and healthy cells (*darker beige*) (**h**), as well as **i**–**l** improving safety control using suicide switch or on-switch. Detailed mechanisms were further illustrated in the section “Overcoming Challenges with Smarter CAR Designs”

Different from B cell malignancies, the application of the CAR-T cell strategy to non-hematopoietic cancer is faced with physical barriers as well as a variety of approaches that tumors employ to blunt host immune-surveillance [49]. Therefore, obstacle factors existing in current CAR-T trials such as fibrosis, inflammation, autoimmunity, T cell exhaustion and persistence, or recurrence must be overcome with smarter redirected T cell designs to achieve optimal therapeutic results (Fig. 3).

### Enhancing trafficking

Tumors are organized tissues with numerous connections with surrounding cells [50], including resident immune system, i.e., myeloid compartment and lymphoid compartment [51], vasculature, fibroblasts, signaling molecules, and the extracellular matrix [52]. In addition, the low pH, low oxygen, low nutrients condition, and the tumor surrounding tissues, especially blood vessels and fibroblasts, make therapeutic delivery cruelly difficult [53].

In current trials, homing of CAR-T cells to the tumor sites is often inefficient owing to the reasons mentioned above. The successful application of CAR-T cells against hematological malignancies is at least partially attributed to the fact that tumor and effector T cells tended to migrate to similar sites due to their shared hematopoietic origins. Solid tumors are known to secrete immuno-factors, including chemokine, cytokine, and growth factors, preventing effector T cells from infiltrating into the tumor bed (Fig. 3a). In 2010, a recombinant chemokine receptor ligand CCR2(b) was introduced into CAR-T cells targeting MSLN [54], and GD2 [55], because the endogenous chemokine receptors on T cells adequately mismatch with the chemokines secreted by the tumors. Beyond minimizing the effects of chemokine, strategies targeting activated surrounding fibroblast (fibroblast activation protein, FAP) in mouse models of mesothelioma and lung cancer [56, 57], vasculature (anti-VEGFR-2) in melanoma, and renal cancer (NCT01218867) [58], as well as tumor stroma (heparanase) in neuroblastoma mice [59], have been tested.

Insufficient T cell trafficking is a major functional challenge in anti-cancer immunotherapy. Little has been improved regarding T cell infiltration in the past decade, which is primarily due to our limited understanding of the tumor surrounding compartments and their effects on CAR-T cells [51]. More tumor-associated biomarkers related to T cell filtration should be investigated, such as alpha smooth muscle actin, Thy-1, desmin, and S100A4 protein [60]. In addition, strategies developed in non-CAR-T therapy context might also be beneficial to CAR-T design and utilized for penetrating the physical barrier and thus enhancing CAR-T cell trafficking. Beyer et al. described a self-dimerizing epithelial intercellular junction opener JO-1 that bound to desmoglein 2 [61]. Manipulation of JO-1 transiently broke tight junctions in polarized epithelial tumor cells, resulting in increased efficiency in mAb treatments (trastuzumab and cetuximab) in xenograft tumor models. Moreover, intratumoral relaxin expression was reported to degrade extracellular matrix protein around solid tumors transiently, which enhanced trastuzumab treatment [62] (Fig. 3b). Non-signaling extracellular hinge domain of CAR-T has also been shown to exhibit an impact on the migratory capacity of CAR-T cells. Qin et al. generated two versions of CAR vectors, with or without a hinge domain, targeting hematopoietic, and solid tumor antigens [63]. The CARs with a hinge domain demonstrated better expansion and migration capacity in vitro and CAR-T cells expressing anti-mesothelin CARs containing a hinge domain showed enhanced antitumor activities.

### Reforming microenvironment

Malignant transformation and the growth of tumor mass influence surrounding microenvironment, which subsequently gives rise to peripheral immune tolerance [64]. In return, tumor microenvironment provides a driving force contributing to not only tumor invasion and metastasis but also pharmacokinetic and pharmacodynamics resistance [65].

In studies targeting solid tumors, CAR-T cells surmount challenges conferred by anti-inflammatory factors (e.g., TGF-β and IL-10), immune suppressor cells (Tregs, Bregs, myeloid cells, neutrophils, macrophages), antigen loss, tissue-specific alterations, etc., [51, 64]. Even if CAR-T cells successfully trafficked to the tumor sites, tumor microenvironment might suppress or even inactivate them. Therefore, with the aim to equip CAR-T cells with capabilities to remodel the suppressive tumor microenvironment by secreting anti-cancer cytokines, strategies named TRUCK have been extensively utilized in CAR-T studies (Fig. 3c). To date, several cytokines have been adopted into TRUCK designs, including IL-12 [66, 67], IL-18 [68], and TNFRSF14 [69]. Recently, a leading-edge TRUCK system using synNotch receptor was described by Roybal et al. [70, 71], which was engineered to produce a range of specific payloads in response to the target antigens. In addition to inflammatory cytokines, the flexible synNotch system could also express pro-tumor cytokine antibodies, checkpoint antibodies, bispecific antibodies, and adjuvants. Using synNotch TRUCK system, more antitumor cytokines or other factors with the potential to reform the local environment based on the specific tumor heterogeneity might be exploited. For instance, reported as potential anti-cancer therapeutic candidates [72], cytokines such as IL-24 should be further tested.

### Anti-immunosuppression

Immunosuppression is a great challenge to effective CAR-T therapy, as it enables the tumor cell to escape from antitumor immune responses [73]. Various types of strategy have been exploited to engineer CAR-T cells to fight tumor immunosuppression. Manipulation of the endogenous TCR/MHC (Fig. 3d) and induction of additional immune checkpoint receptors (Fig. 3e) are two promising strategies investigated extensively.

Immune escape mediated by inhibitory pathways via the interaction of activated killer T cell receptors with their ligands, such as programmed cell death1 (PD-1) [74], and cytotoxic T lymphocyte antigen 4 (CTLA-4) on T cells is another major factor [75]. To overcome repressive solid tumor environments and enhance the activity and persistence of CAR-T cells, combined therapies using co-administration of immune checkpoint inhibitors or cytokines with CAR-T cells have been employed. Immune checkpoint inhibitors block the immune checkpoint pathways by targeting key regulators in the pathways (e.g., PD-1/PD-L1, CTLA4) to enhance the immune activity of patients’ effector T cells [76]. The addition of anti-PD1 monoclonal antibody has been shown to mute the inhibitory effect of such receptors and enhance the function of CAR-T cells in preclinical models [77–79]. Many groups are now attempting to generate CAR-T cells resistant to PD1-PDL1 and CTLA4-CD80/CD86 signaling [80], and some of these CAR constructs are currently under investigation in clinical trials as discussed in the previous section (Table 1).

Immunosuppressive soluble factors, like TGF-β and IL10, have been demonstrated to inhibit CAR-T cell activities [81]. Specifically, TGF-β has direct negative effects on T cell differentiation and cytotoxic function, thus hampering T cell effector functions [82]. TGF-β and IL10 can also inhibit antigen-presenting cells, leading to hampered activation of tumor-reactive T cells [83]. Other factors including prostaglandin E2 (PGE2) and adenosine have also been demonstrated to inhibit T cell proliferation and differentiation via signaling through G-coupled receptors [84, 85]. CAR-T cells can be potentially engineered to include the expression of a dominant-negative form of the receptor of these factors to overcome their inhibitory effects [86].

In addition to the soluble immunosuppressive factors, various suppressive surveilling immune cells within the tumor microenvironment, such as Tregs, myeloid-derived suppressor cell (MDSC), and tumor-associated macrophage (TAM)/tumor-associated neutrophils (TAN) with the so-called M2 and N2 phenotype, present another obstacle against successful CAR-T treatment. Tregs have been shown to inhibit T cell activity through cell-to-cell contact inhibition and via soluble factors such as TGF-β and IL10 [87]. Tregs hamper T cell activity by producing TGF-β, IL10, and also other suppressive agents like IL35 and adenosine [88]. MDSC, M2-TAM, and N2-TAN inhibit antitumor immune response by producing TGF-β, PGE2, reactive oxygen/nitrogen species, and arginase [89, 90]. M2-TAM can express high levels of PD-L1, which can interact with PD1 on CAR-T cells and inhibit them [91]. To overcome the effect of such suppressive surveilling immune cells, the CAR-T design has been proposed to target both the tumor cells and immune cells. Ruella et al. demonstrated the feasibility of targeting CD123-positive Hodgkin lymphoma cells and TAM [92]. However, whether this strategy is universally applicable to other malignancies, especially for the solid tumor, still needs to be further tested.

### Elevating recognition specificity

The genetic heterogeneity of solid tumors is a major issue to treat such malignancies with CAR-T. Unlike CD19 in B cell leukemia, there is no such “panacea” antigen available for solid tumors. Worse still, cross-reactions (i.e., “OFF target” and “ON target OFF tumor”) with bystander non-tumor cells have been widely observed in CAR-T studies, leading to severe or even lethal adverse effects caused by T cells attack to the essential tissues.

This off-tumor toxicity could be restrained by designing CAR-T cells with enhanced specificity using two or more extracellular antigen recognition motifs. Presently, three major classes of bispecific CARs have been employed in T cell engineering, namely, dual CAR (Fig. 3f), tandem CAR (TanCAR) (Fig. 3g), and inhibitory CAR (iCAR) (Fig. 3h). Kloss et al. firstly reported co-expression of a suboptimal CAR with an additional chimeric costimulatory receptor (CCR), which sufficiently recognized and killed cells expressing the two antigens [93]. Dual T cells would be fully activated only when the two target antigens present simultaneously on tumor cell surface, which significantly strengthened the specificity and thus leaving the bystander cell untouched. Such dual CAR design was reported to generate specific toxicity towards tumor cells [94]. Recently, an advanced AND-gate circuit using synNotch receptors further upgraded the dual CAR system [70, 95]. Upon activation by associating with the first antigen, synNotch system induced a secondary CAR expression via intracellular transcriptional domain to modulate T cell activity in the presence of the second antigen. Interestingly, initially designed as an AND-gate circuit, a TanCAR that linked two distinct scFVs was adopted in treatments against HER2-positive glioblastoma [96, 97], yet has been reported to function as an OR-gate circuit [98], which could kill either CD19-positive or CD20-positive leukemic cells in vivo. This TanCAR has been proved to be particularly useful in the clinic to prevent resistance caused by loss of antigens. To further minimize the T cell “on-target off-tumor” activity towards normal tissues, a NOT-gate circuit using a killing CAR and an inhibitory CAR was developed [99].

### Improving safety and control

Most of the efforts at CAR-T engineering has been devoted to improve the strength of clinical response and to prolong T cell proliferation and persistence. However, the adverse effects observed in human trials suggest the urgency of additional consideration on safety control mechanisms. Utilization of control systems should be regarded as the priority in the next generation CAR-T therapy design as T cells are autonomous without regulatory mechanisms.

To date, a growing number of user-control systems have been developed to modulate CAR-T cell expression. In 1997, Bonini et al. firstly reported successful control of graft-versus-host disease (GVHD) using a suicide gene named herpes simplex virus thymidine kinase (HSV-tk) in adoptive T cell therapy, which rendered the T cells susceptible to ganciclovir treatment [100]. In 2001, Fas intracellular domain (ΔFas) was applied as a T cell suicide switch to combat GVHD in marrow transplantation [101]. In 2011, Di Stasi and colleagues introduced a human caspase-9 (iCasp9) as an off-switch to conditionally trigger apoptosis of CAR-T cells by dimerization upon the treatment of small molecules (Fig. 3i) [102]. In hematopoietic stem cell transplantation recipients, 90% of iCasp9-modified T cells could be eliminated within 30 min under AP1903 administration without severe adverse effects or recurrence [103]. Synthetic death control switches are likely to be extensively utilized in future adoptive immunotherapy owing to their high effectiveness, easy controllability, short time of on-set, and mild adverse effects, despite that minority of the T cell populations could escape from the apoptotic signal, resulting in persistent cytotoxicity [103]. Alternatively, CAR-T cells could be removed via apoptosis using antigen-specific monoclonal antibodies (Fig. 3j), such as rituximab for CD20 epitope [104], and cetuximab for EGFR epitope [105]. In addition to selective elimination, expression of previously known antigens enables tracking of CAR-T cells in vivo.

In contrast to the death switch, a distinct class of on-control mechanisms is considered as safer manipulating strategies, as CAR-T cells using on-switches are defaulted to be unresponsive. In this design, CARs are conditionally expressed or form a functional structure in the presence of the inducer, removal of which terminates the transcription or inactivates CARs. Wu et al. described an advanced on-switch CAR using a split construct in vivo (Fig. 3k). The split CAR design distributed into two separate polypeptides, an extracellular binding domain scFv and a downstream signaling element ITAM, two parts reassembled and activated under the treatment of a small molecule rapalog [106]. In 2016, Morsut and colleagues further developed a tissue ligand-specific on-switch CAR construct based on synNotch receptors using a tetracycline (Tet)-regulated promoter (Fig. 3l) [107]. It is noteworthy that expression levels of CARs are generally dose-dependent before saturation, which allows manipulating the strength of CAR expression accordingly. In Tet-on systems, CARs still exist after removal of the inducer; hence, it requires further depletion to eliminate CAR-T cells ultimately. At this stage, an antibody like myc could be conveniently applied to target the tagged CARs as a safeguard factors after adoptive transfer [108]. In principle, integration of the on- and off-switch systems enables us to turn on the expression of CARs at the wanted time point, to turn off when the doses are sufficient, and to erase T cells from patients once the treatments are completed.

Strategies involving codon optimization [109–111] or construct alteration using lentivirus [112], transposon [113], or RNA [114] were also employed in the next generation CAR design to ameliorate side effects resulted from T cell overactivation. Hopefully, a combination of suicide/on switches, optimized coding, relevant downstream feedback response, and sensor circuits in the future trials could help to establish global control systems to modulate the expression, strength, and timing of the engineered CAR-T cells.

## Conclusion

CAR-T has demonstrated itself as a promising treatment for the solid tumor in preclinical and clinical studies. Progress in the following aspects of CAR-T should facilitate the development of the therapy. First, CAR designs need to be further optimized to give better T cell activation, recognition specificity, antitumor activity, and safety control. The search for optimal signaling and costimulatory domains will continue to improve the efficacy of CAR-T therapy. Application of bispecific CAR is a promising way to enhance the tumor cell recognition specificity, limiting unexpected attack to the normal cells. Because of the genetic heterogeneity, personalized modification during CAR construction might be needed to deliver the maximum antitumor effect. Second, identification of the most suitable T cell subset for genetic engineering and the establishment of a standard ex vivo T cell processing procedure are critical for producing long-lasting CAR-T cell persistence and memory for optimized antitumor response. Additional modifications to CAR-modified T cells might be necessary to overcome immunosuppressive tumor microenvironments. Strategies like the introduction of anti-cancer cytokines (IL12), manipulation of the immune checkpoint signaling (PD1/CTLA4), immunosuppressive soluble factors (TGF-β, IL10), and suppressive surveilling immune cells are aspects worth to be explored. Third, the establishment of standard clinical protocols is needed. Improvements in the CAR-T cell itself will require parallel developments in clinical protocol design, including patient preconditioning, cytokine support, and other potential co-administered treatments. Preconditioning of the patient before adoptive cell therapy is thought to have a significant effect on the immune response, thereby producing a potential therapeutic window for CAR-T cell activity. Improvements in CAR design and better understating of the interaction between tumor and immune system will help to overcome the hurdles currently limiting the application CAR-T in solid tumor treatment.

## Abbreviations

ALL: Acute lymphoblastic leukemia
BTC: Biliary tract cancer
CAIX: Carboxyanhydrase-IX
CARs: Chimeric antigen receptors
CAR-T cells: Chimeric antigen receptor T cells
CCA: Cholangiocarcinoma
CCR: Chimeric costimulatory receptor
CEA: Carcinoembryonic antigen
cMET: Hepatocyte growth factor receptor
CTLA-4: Cytotoxic T-lymphocyte antigen 4
EGFR: Epidermal growth factor receptor
EpCAM: Epithelial cell adhesion molecule
EphA2: EPH receptor A2
FAP: Fibroblast activation protein α
FDA: Food and Drug Administration
FR: Folate receptor
GD2: Disialoganglioside
GPC3: Glypican-3
GVHD: Graft-versus-host disease
HER2: Human epidermal growth factor receptor-2
HSV-tk: Herpes simplex virus thymidine kinase
iCAR: Inhibitory CAR
IL: Interleukin
L1-CAM: L1 cell adhesion molecule
MDSC: Myeloid-derived suppressor cell
MPM: Malignant pleural mesothelioma
MSLN: Mesothelin
MUC1: Mucin
NK cells: Natural killer cells
NSCLC: Non-small-cell lung carcinoma
PD-1: Programmed cell death-1
PDAC: Pancreatic ductal adenocarcinoma
PGE2: Prostaglandin E2
PR: Partial response
PSMA: Prostate-specific membrane antigen
ROR1: Receptor tyrosine kinase-like orphan receptor 1
scFV: Single chain variable fragments
TAA: Tumor-associated antigens
TAM: Tumor-associated macrophage
TAN: Tumor-associated neutrophils
TCR: T cell receptor
Tet: Tetracycline
Treg cells: Regulatory T cells
TRUCK: T cell redirected for universal cytokine-mediated killing
VEGFR: Vascular endothelial growth factor receptor
